# Incidence of Surgical Site Infections in Multicenter Study—Implications for Surveillance Practice and Organization

**DOI:** 10.3390/ijerph18105374

**Published:** 2021-05-18

**Authors:** Anna Różańska, Jerzy Rosiński, Andrzej Jarynowski, Katarzyna Baranowska-Tateno, Małgorzata Siewierska, Jadwiga Wójkowska-Mach, Polish Society of Hospital Infections Team

**Affiliations:** 1 Chair of Microbiology, Jagiellonian University Medical College, Czysta Str. 18, 31-121 Krakow, Poland; mbmach@cyf-kr.edu.pl; 2 Institute of Economics, Finance and Management, Faculty of Management and Social Communication, Jagiellonian University, 30-348 Krakow, Poland; jerzy.rosinski@uj.edu.pl; 3 Interdisciplinary Research Institute, 67-200 Głogów, Poland; ajarynowski@gmail.com; 4 District Hospital in Bochnia, 32-700 Bochnia, Poland; baranowska1@gmail.com; 5 St. Rose Hospital, 30-394 Krakow, Poland; tsiewi@wp.pl

**Keywords:** surgical site infections, infection control and prevention, surveillance

## Abstract

Introduction: WHO core components of healthcare-associated infections (HAIs) prevention and control include their surveillance system. In Poland, there are no widespread multi-center infection surveillance networks based on continuous, targeted, active methodology. One of the most important form of HAIs are surgical site infections (SSIs). The aim of this study was to analyze the incidence of SSIs, in the context of seasonal differentiation. Seasonal differentiation could be connected with weather conditions, but it also can be affected by personnel absence due to holidays and furlough. The second aspect may influence organization of work and increased absenteeism may contribute to lowering the quality of patient care. Healthcare associated infections are the phenomenon which can be especially affected by such factors. Methods: The data used originate from the targeted, active surveillance reports obtained from the six years period, based on the ECDC recommendations. Results: Highest incidence rates of SSIs were found after operations performed in June and August, equal to 1.8% and 1.5% respectively and the lowest in October was 0.8%. These differences were statistically significant: for June incidence: OR 1.6, 95% CI 1.03–2.5, *p* = 0.015. Another approach showed a significant difference between the level of incidence in the period from November to January together with from June to August (1.35%), comparing to the rest of the year (1.05%). Also the rates of enterococcal and Enterobacterales infections were significantly higher for the period comprising months from November till January and from June to August. In Poland these are periods of increased number of absences associated with summer, national and religious holidays. Conclusions: Our results show that the short-term surveillance data limited to several days or months are not sufficient to obtain a valuable description of the epidemiological situation due to HAI. Efforts should be undertaken in order to implement wide net of hospital acquired infections, including SSI on the country level.

## 1. Introduction

Healthcare-associated infections (HAI) surveillance system is listed among WHO core components of HAI prevention and control in acute hospitals at facility and national level. European Centre for Disease Prevention and Control (ECDC) is a coordinating body in Europe for developing surveillance strategies, including detailed guidelines [1]. Among ECDC modules of infection surveillance, there are recurrent point prevalence study (PPS), two different modules for active surveillance (in intensive care units and for surgical site infections) and additionally *Clostridioides difficile* infections [2]. Methodology of the PPS study is based on prevalence, i.e., describing the number of infections at a certain point in time (a short period). In most European countries participating in the project, the study is repeated every few years, or as in Poland—every year. Protocols of HAI-Net SSI and HAI-Net ICU, allow for submission of data gathered for at least three months, chosen arbitrarily by hospitals [3]. According to WHO, all surveillance systems should rely on good data quality, which includes the appropriate application of case definitions and good microbiological laboratory procedures. And the proper usage of such registration tools determine obtaining credible data for hospital epidemiology description [1].

In Poland, there are no widespread multi-center infection surveillance networks based on continuous, targeted, active methodology. Data about HAIs are based mainly on the results of the PPS project [4]. The HAI surveillance program, based on ECDC HAI-Net SSI and ICU protocols, coordinated by the non-governmental Polish Society of Hospital Infections, was implemented in 2013 in several hospitals. Hospitals participated in this program voluntarily. Surgical procedures for SSI surveillance were chosen by each hospital, depending on the hospital profile and needs. As a result, among monitored procedures there were those recommended by ECDC (like cesarean section, hip and knee arthroplasty, cholecystectomy, laminectomy etc.) and others, such as thoracic surgery, gynecology and urology procedures, and broad spectrum of musculoskeletal procedures. The results of surveillance after cesarean section and thoracic procedures indicating some specific problems in Polish hospitals have been already published [5,6].

Surgical site infections are one of the most important form of HAIs, accounting for a quarter of the total of HAIs [7]. The occurrence of SSI results in additional costs for hospitals, patients and healthcare systems. According to Broex et al. in European hospitals patients with SSI constitute a financial burden approximately double than corresponding values for patients without this complication [8]. Badia et al. based on a systematic review of papers from six European countries stated, that the length of stay in hospitals was higher among patients who developed an SSI relative to uninfected ones and that it was true for all studies [9]. Additionally, they founded the highest number of days required in hospital following the development of an SSI after orthopedic and trauma surgery in the UK—an additional 54 days with an SSI [9]. In Poland, just single studies of the burden of hospital acquired infections were performed so far [10,11]. In the light of the burden of SSIs, it is certainly disturbing that the authors of studies on the epidemiology of SSI in a specific, narrow population of patients reported even several times higher rates in Polish hospitals compared to European [12,13]

The results of some studies, not very numerous, indicate that the risk of HAI is seasonal-dependent—higher in warm months [14,15,16,17,18,19]. However, we also know that indicators such as the number of full time nurses or physicians probably influence the effectiveness of infection control and prevention procedures [20,21].

The aim of this study was to analyze the incidence of SSI and their etiology in the context of seasonal differentiation. It was hoped that in result it would be possible to obtain the data necessary to identify intervention needs and to optimize the management of infection surveillance—including prevention and control practices during the calendar year.

## 2. Materials and Methods

The data used in this publication originate from the Polish Society of Hospital Infections program database of active registration of healthcare associated-infections and are related to SSIs reported by five Polish hospitals from 1 January 2013, until 30 June 2018.

The participation in the programme by hospitals was voluntary, and the analyzed databases were anonymized at the facility level. This work was approved by the Bioethics Committee of Jagiellonian University (No. KBET/122.6120.118.2016 from 25 May 2016). The study was based on the data gathered during routine caretaking and the analysis did not include any individual participants’ data. As a result no statements of consent from participants were required. The study in its present form was approved by the local Bioethics Committee of Jagiellonian University.

All study hospitals had infection control teams consisting of infection control nurses (no more than one per 200 beds) and a physician as the team leader (the duties related to the study averaged 1/5 of their total workload). SSI were identified based on ECDC definitions [3] and by taking into account the time of symptom onset, i.e., whether symptoms occurred within 30 days following the surgical procedure or within 90 days if an implant was in place (only in some procedures in musculoskeletal system—arthroplasty of hip, craniotomy and hernia among others). Surgical site infections may occur and are classified in three different forms: superficial, deep incisional and organ/space. ECDC protocol provides detailed definitions for all three types. Superficial infection occurs within 30 days after the operation and involves only skin and subcutaneous tissue of the incision and at least one of the following: purulent drainage with or without laboratory confirmation, from the superficial incision; organisms isolated from an aseptically obtained culture of fluid or tissue from the superficial incision; at least one of the following signs or symptoms of infection: pain or tenderness, localized swelling, redness, or heat and superficial incision is deliberately opened by surgeon, unless incision is culture-negative; diagnosis of superficial incisional SSI made by a surgeon or attending physician. Deep incisional infection occurs within 30 days after the operation if no implant is left in place or within 90 days if implant is in place and the infection appears to be related to the operation and infection involves deep soft tissue (e.g., fascia, muscle) of the incision and at least one of the following: purulent drainage from the deep incision but not from the organ/space component of the surgical site; a deep incision spontaneously dehisces or is deliberately opened by a surgeon when the patient has at least one of the following signs or symptoms: fever (>38 °C), localized pain or tenderness, unless incision is culture-negative; an abscess or other evidence of infection involving the deep incision is found on direct examination, during reoperation, or by histopathologic or radiologic examination; diagnosis of deep incisional SSI made by a surgeon or attending physician. The organ/space infection occurs within 30 days after the operation if no implant is left in place or within 90 days if implant is in place and the infection appears to be related to the operation and infection involves any part of the anatomy (e.g., organs and spaces) other than the incision that was opened or manipulated during an operation and at least one of the following: purulent drainage from a drain that is placed through a stab wound into the organ/space; organisms isolated from an aseptically obtained culture of fluid or tissue in the organ/space; an abscess or other evidence of infection involving the organ/space that is found on direct examination, during reoperation, or by histopathologic or radiologic examination; diagnosis of organ/space SSI made by a surgeon or attending physician [3].

Targeted active surveillance was carried out using a standardized research protocol based on uniform criteria and definitions for diagnosing infections, in accordance with the ECDC guidelines [3]. For all surgeries, the following data characterizing the procedure and the patient were registered: age, date of hospital admission and date of surgery; ICD-9 code of the procedure and others. For SSIs diagnosis, aside from the data mentioned above, the following information was also collected: date of discharge from hospital, date of first infection symptoms, whether the infection was confirmed microbiologically and—in case of confirmation—e.g., microbiology, time of diagnosis (before discharge, post discharge, re-hospitalization) and SSI type (superficial, deep, or organ/space). No study hospitals actively performed a surveillance of SSIs post-discharge, SSIs were registered only if patients reported symptoms during visits to the hospital out-patient unit, but without active cooperation with ambulatory care staff (or via telephone interview with patients). Microbiological culture-based tests were performed when ordered by the attending physician. However, the method used for taking samples included swabs without a collection system to identify anaerobic bacteria. When analyzing the relationship between seasonality of SSIs, i.e., the time of surgery in months and microbial etiology, a division into several microbial groups—staphylococci, enterococci, the Enterobacteriaceae family and others—was adopted.

Surveillance of SSI was conducted both for type of surgical procedures included in ECDC HAI-Net protocol, as well as those resulting from individual decisions of hospitals and infection control teams. For analysis, four categories of surgical procedures were created:

1. General and gastrointestinal surgery: appendectomy, cholecystectomy, surgery on the small intestine, surgery on the large intestine (ICD-9 codes 42–54)
2. Urogenital surgery: cesarean section, vaginal hysterectomy, abdominal hysterectomy, prostatectomy, other male and female genital surgery (ICD-9 codes 60–64 and 65–71), kidney, ureter surgery, bladder surgery, urethral surgery (ICD-9 codes 55–59),
3. Operations in the musculoskeletal system: limb amputation, laminectomy, knee arthroplasty, hip arthroplasty, lower leg fracture, open fracture, craniotomy, other musculoskeletal surgery (ICD-9 codes 76–84)
4. Other surgery, not present in the above categories.

The analysis aiming to show a possible seasonal variation was carried out using 2 models: model A compared the incidence rates in individual months of the year, while model B searched for a trend in the incidence rates without a predetermined criterion.

The studied hospitals did not perform a compliance measurement of prevention procedures and a validation of SSIs surveillance. None of the hospitals reported staff absence data prospectively, this type of data was not routinely or otherwise available to the infection control teams under study. The data mentioned above were sourced from operating theatre documentation, which proves that only real information was added to the database. 

## 3. Statistical Analyses

The aim of the analysis was to explore seasonal patterns of SSI, expressed by incidence rates and additionally grouped by etiology. Principles of data collection was previously described [5]. For All 33,467 hospitalization records, operation date have been filled. In 69 records, a proper ICD-9 code was not provided, so these procedures were classified as other according to operation place. Among 477 all registered SSI, 403 have given date and etiology 74 SSI were excluded due lack of knowledge of microbiological agent or date. The two-sided z-test was used to compare two-sample incidences (observed proportions) with the Yates continuity correction using the R programming environment. The lowest incidence for October was chosen as a reference level to test equality of given incidences in the months of year. On the other hand, we compared holiday vs. non-holiday samples against each other with the same z-test, taking also etiology into account. *p*-value less than 0.05 suggests that there is a significant difference between compared incidences for given periods.

## 4. Results

This study encompassed 33,467 surgery patients at five Polish hospitals of different profiles, size, and ownership. Women constituted the majority of this group—65% of total number of surgery, and similarly in case of SSIs—61%. However, no significant differences were observed for incidence between men and women. Dominance of women in the study population was connected with the fact, that most of the procedures were operations in the genitourinary system (12,911, 38.6% of all)—mainly cesarean section and hysterectomy and as a result of which 178 SSIs (37.3% of all) were detected. The other numerous procedures were musculoskeletal (12,453, 37.2% of all), and 138 SSIs (28.9%) were detected accordingly. Domination of genitourinary system procedures and cesarean section was reflected in the median age of women in this category—30 years, compering to respective value for men—66 years. For other categories the median age values for men and women were similar. In case of musculoskeletal procedures they were 61 and 66, for digestive tract—54 and 55 respectively. Median age for men and women in case of “other surgery” category was the same—63.

The lowest incidence rate was related to musculoskeletal procedures, 1.1%, while the highest, 2.6%, among patients undergoing various types of operations included in this work as category no. 4, which constituted only 12.9% of the procedures under surveillance. Almost half of SSI were deep incisional type—46%. Superficial infections accounted for a slightly smaller share—38%. Organ space SSIs accounted for 11% and in case of 20 infections data on the type were missing.

In the absence of differentiation in the number of operations performed in the examined periods of the year, highest incidence rates of SSIs were found after operations performed in June and August, equal to 1.6% and 1.83% respectively and the lowest in October was 0.8% (Table 1). These differences were statistically significant: for June incidence OR 1.6, 95% CI 1.03–2.5, *p* = 0.015. (Table 1).

An analysis taking into account an additional factor, i.e., the division of the calendar year into two separate periods, that is one from June till August together with from November till January compared with the rest months of the year showed a significant difference in the level of incidence, Additionally, the incidence rates values for Enterobacteriales and enterococci during increased absenteeism were 0.44% and 0.20%, respectively, while in the remaining months—0.26% and 0.12% and the difference was statistically significant —Table 2. In Europe June and August are the typical holiday months in which employees go on holidays, as well as in Poland, although in Poland November, December and January are of the months of increased number of absences associated with national or religious holidays. Among those there are the so-called long weekends in Poland: All Saints’ Day on 1st and Independence Day on 11 November; Christmas and the New Year break in December (but usually days before those days are not working), which lasts up to at least 6 January. 

## 5. Discussion

The correlation between season and SSIs incidence is well defined in many specialties, but the obtained results indicate a problem concerning infection control in terms of the organization of personnel work—a factor not related in any way to the generally understood problems and conditioning of infection prevention and control run by infection control teams. Our results showed morbidity in months with potentially high absenteeism (June to August and November till January) higher by one third than in the rest of the year, in addition, infections caused by Enterobacteriales were two times more frequent.

On the one hand, the results of this study confirm a very universal problem of higher incidence in case of hospitalization or infections treatments performed in warm months and described in many regions of the world [14,15,16,17,18,19], but they also indicate an additional element that significantly increases the risk of SSIs, i.e., conducting operations during public holidays, summer or seasonal holidays and religious celebrations. It is likely that the significantly higher incidence rate results from problems in the work organization of the ward or operating theater, including increased workload and current obligations of the staff who remained at work. This situation may be the result of a broader process—the functioning of the institution as an organization with a specific culture that tends to be favourable towards the needs of people higher in the hierarchy—whether those needs be formal or informal. Such a culture would simultaneously depreciate the needs of people in lower positions [22]. Using the typology of F. Laloux [23], we can predict two types of organizational cultures. If the organization places employees situated higher in structure and/or those in power higher in the hierarchy (academic titles, official position) then repeatability of behavior and conformity can be expected. Hence, insufficient staffing (concerning the number of employees or their qualifications) will be accepted if they recur cyclically at specific periods of the year and the needs of people in the hierarchy are also secured (this is called “amber” culture).

The so-called orange culture is characterized as being guided by the principle of meritocracy, i.e., promotion of people with high competences needed by the company; it gives a high value to specialists involved in the effective functioning of the organization. Unfortunately, such organization culture—one that focuses on the effectiveness, efficiency and competence of staff—does not guarantee a high level of stability in HAIs control and prevention either. Unfortunately, the organizational culture change—from “orange” to „amber”—appears to be a challenging target for IPC teams [24]. That is because the interests of experts, e.g., highly qualified doctors in the specialty market, are the top priority. In practice this means that those experts—surgeons and anesthesiologists are a typical example in Polish hospitals—can easily change jobs and leave the current facility. Therefore, this kind of organizational culture is going to result in a lack of protection for the patient’s safety: on days inconvenient for the best specialists, it will be expected of interns and novice nurses—who are placed at the bottom of the hierarchy—to remain on duty. Similar problems also apply to other groups of medical professionals, among others ICU setting staff [24] or diagnosticians [25].

This observation should have practical implications and should be taken into account by infection control teams and ward staff in setting procedures and management—especially considering the difficulties in implementing effective teamwork in interdisciplinary teams, which is a big challenge for health professionals working in acute hospital [26]. Overcoming weaknesses or even dangerous practices in both cultures is associated with the introduction of labor standards and the effective presence of specialists. In other words, a legal framework should be created with the aim to “force” a specific behavior in the organization. With the current staff resources, this may be a difficult standard to maintain. However, it is worth remembering that the requirements that are kept in the form of records independent of the will of the parties are not only descriptive (what the work should look like), but also normative (rewarded, expected/penalised, undesirable behavior). Even if it is not possible to ensure a full implementation of such standards at this time, we have a clear model for the institution’s aspirations. It seems that determining the work standard and employee presence will improve the functioning of both organizational cultures described. Amber-like organizations take legal regulations extremely seriously and often have the primacy of correctness of the process (described by legal acts) over the results achieved. Therefore, the new process description will be strongly binding on these organizations. In turn, “orange” cultures can direct their activities towards achieving results (e.g., implementing medical procedures) at minimal costs. Hence, for these organizations, external legal regulations are normalizing the “overly creative” approach to achieving results. In practice, implementation of multidisciplinary team-based leadership training program for health professionals in pre- and post-diploma education can also serve as catalysts for health improvement efforts in resource-limited environments should be helpful [27], like in Poland.

Many previous studies have shown a significant increase in incidence in the summer months, not only for SSI, but also for blood or urinary tract infections, in particular those caused by Gram-negative rods [28]. The justification of the increase in infections due to Gram-negative rods in the summer includes temperature, humidity, and human behavior (e.g., dietary habits and recreational activities). Similar observations of higher prevalence in the warm season were also observed for SA infections, especially for skin and soft tissue infections [29]. In our study, in addition to significant differences in incidence in the November-January and June-August periods (i.e., recognized by the authors as periods of increased absenteeism associated with different kinds of holidays) compared with the remaining months of the year, a higher prevalence of Enterobacteriales bacillis and enterococci isolated from SSI cases was also found. Therefore, the microbial etiology of SSIs confirms our hypothesis about the need to take action to improve patient safety and reduce the impact of the work organization on the risk of SSI development, including infections of Enterobacteriales bacillis etiology, which carry a significant risk of drug resistance, and thus therapeutic failures.

The results of our study, besides the seasonal differentiation, also highlight another aspect of infection control, escpecially registration, i.e., the need of hospitals’ participation in the surveillance network grouping many settings or at least several. In our study, not entirely clear significance of differences may be the result of relatively low number of infections in individual months. In case of SSIs, in one hospital the numbers including monthly data, do not exceed a few cases for the procedures with higher incidence. For the procedures with the incidence about 1% or lower, there yearly number of SSI is not greater than a few [5,6,30].

## 6. Limitations

The limitations of our study are self-reported data lacking an external validation, sample size, and the small number of hospitals that participated in the project. However, it is a unique piece of research due to several factors: we must take into account the lack of studies devoted to the relationship between incidence rates and work organization in various hospitals/wards. There is a scarcity of studies aimed at identifying conditions related to periods of increased absenteeism, while being indicative of important aspects of infection surveillance that are worth considering in both daily work and in developing a long-term strategy. Due to the lack of prior research on the topic of expected disturbances in organization of work in surgery units at given periods, such as national holidays or vacancy—our study fills the gap in the literature and highlights the need for further research.

## 7. Conclusions

It seems that the analysis of the epidemiological situation of a particular hospital—either at the regional or national level—by means of prevalence studies, or limiting the registration of selected forms of infection to a three-month period, can lead to false conclusions. This probably applies not only to Polish hospitals, but perhaps to other European countries as well. In Poland, there is no large network/surveillance program based on active, continuous monitoring and registration. The epidemiological situation in the field of HAIs, primarily at the hospital and country level, is assessed mainly on the basis of the results of the PPS survey, which covers one week during a usual working period. Therefore data obtained in this way may be misleading. Undoubtedly, in order to obtain the most reliable data, it is necessary to implement a continuous targeted long-term surveillance program in hospitals.

## Figures and Tables

**Table 1:** Incidence of surgical site infections according to time of detection—two models.

Time Period	Number of Procedures	Number of Surgical Site Infections	Incidence	OR (95% CI)	Z-Test *p*-Value
Period—month, model A					
January	2554	26	1.0%	1.14 (0.7–1.86)	0.458
February	2588	34	1.3%	1.52 (0.96–2.4)	0.691
March	2920	38	1.3%	1.19 (0.74–1.9)	0.710
April	3004	32	1.1%	1.28 (0.81–2.02)	0.559
May	3130	31	1.0%	1.2 (0.75–1.9)	0.332
June	2858	50	1.8%	1.6 (1.03–2.5)	0.015
July	2963	36	1.2%	1.14 (0.71–1.83)	1.000
August	2556	39	1.5%	1.83 (1.18–2.85)	0.183
September	2748	26	1.0%	1.1 (0.67–1.78)	0.267
October	3007	25	0.8%	ref	ref
November	2681	37	1.4%	1.36 (0.86–2.17)	0.479
December	2278	29	1.3%	1.28 (0.79–2.09)	0.848
Periods—model B					
Jan, Jun, Jul, Aug, Nov, Dec	15,890	220	1.5%	1.3 (1.08–1.61)	0.003
Feb, Mar, Apr, May, Sep, Oct	17,397	183	1.1%	ref	
**Total**	**33,467**	**403**	**1.4%**		

OR—Raw Odds ratio between given period and total incidences.

**Table 2:** Etiological factors isolated in surgical site infections according holidays vs. non-holidays periods.

Time Periods	Incidence Number of Infections	Etiological Factors of Infections			
		*Staphylococcus* spp.	*Enterococcus* spp.	Enterobacteriales	Others
Holidays period	1.38%	0.35%	0.20%	0.44%	0.38%
	220	56	33	70	61
Non holidays months	1.05%	0.28%	0.12%	0.26%	0.39%
	183	49	21	45	68
***p*-Value of Z-test between holiday vs. non-holiday period**	**0.001**	**0.19**	**0.04**	**0.003**	**0.93**

## Data Availability

The datasets generated or analyzed during this study are available and can be accessed from Anna Różańska (e-mail: a.rozanska@uj.edu.pl) on a reasonable enquiry.

## Abbreviations

OR	Odds Ratio
95% CI	95% Confidence Interval
*p*-value	probability value
SSI	Surgical Site Infection
